# Analytical Parameters of an Amperometric Glucose Biosensor for Fast Analysis in Food Samples

**DOI:** 10.3390/s17112620

**Published:** 2017-11-14

**Authors:** Margalida Artigues, Jordi Abellà, Sergi Colominas

**Affiliations:** Electrochemical Methods Laboratory—Analytical and Applied Chemistry Department, ETS Institut Químic de Sarrià, Universitat Ramon Llull, Via Augusta, 390, 08017 Barcelona, Spain; margalida.artigues@iqs.edu (M.A.); jordi.abella@iqs.edu (J.A.)

**Keywords:** biosensor, chitosan, glucose oxidase, glucose quantification

## Abstract

Amperometric biosensors based on the use of glucose oxidase (GOx) are able to combine the robustness of electrochemical techniques with the specificity of biological recognition processes. However, very little information can be found in literature about the fundamental analytical parameters of these sensors. In this work, the analytical behavior of an amperometric biosensor based on the immobilization of GOx using a hydrogel (Chitosan) onto highly ordered titanium dioxide nanotube arrays (TiO_2_NTAs) has been evaluated. The GOx–Chitosan/TiO_2_NTAs biosensor showed a sensitivity of 5.46 μA·mM^−1^ with a linear range from 0.3 to 1.5 mM; its fundamental analytical parameters were studied using a commercial soft drink. The obtained results proved sufficient repeatability (RSD = 1.9%), reproducibility (RSD = 2.5%), accuracy (95–105% recovery), and robustness (RSD = 3.3%). Furthermore, no significant interferences from fructose, ascorbic acid and citric acid were obtained. In addition, the storage stability was further examined, after 30 days, the GOx–Chitosan/TiO_2_NTAs biosensor retained 85% of its initial current response. Finally, the glucose content of different food samples was measured using the biosensor and compared with the respective HPLC value. In the worst scenario, a deviation smaller than 10% was obtained among the 20 samples evaluated.

## 1. Introduction

The high sugar consumption in the modern diet has been commonly associated with chronic health consequences including risk of obesity, diabetes, cardiovascular disease, and fatty liver disease [1]. Nowadays, two out of three adults and one out of three children in the United States are overweight or obese [2,3] due to the high level of sugar consumption. In the same way, in the European Union 52% of the adult population is now overweight, of which 17% is obese and more than 15% of adolescents in southern European countries are obese [4].

It is also worth mentioning that in the food industry more manufacturing processes are used routinely, and more sophisticated quality control methods are needed to ensure that every ingredient maintains its quality and safety through all processing stages. As a result, analytes like sugars, alcohols, phenols, oligonucleotides, and O_2_ need to be measured at multiple stages of the production process and also in the final product. Moreover, the increasing number of food safety regulations related to alimentary allergens and contaminants require extensive control of different analytes, such as lactose [5] or glutamate [6] among others [7].

Numerous methods are reported for these analyses in food [8,9,10,11]; among them, Colorimetry and High-Performance Liquid Chromatography (HPLC) are well-established and accepted tools for glucose determination in food matrices. However, most of the adopted methods are time consuming, expensive and require specialized personnel. In addition, the presence of interfering species in the sample matrix implies complex sample preparation processes, such as solid phase extraction (SPE) or chemical modifications (e.g., fluorescent functionalization). These sample treatments increase the complexity, time, and cost of the analysis. Therefore, development of fast, cheap, practical, and selective methods for detecting glucose in food is still a research area. In this context, electrochemical biosensors have attracted much attention due to their promising characteristics that fulfill these market demands. Moreover, due to their outstanding features, sensors presenting nano-engineered electrochemical interfaces have gained interest.

Leland C. Clark Jr. and Champ Lyons introduced in 1962 the principle of the first enzyme electrode with immobilized glucose oxidase [12]. Following some improvements, the biosensor was launched onto the market in 1975 by the Yellow Springs Instruments Co. (Yellow Springs, OH, USA). This device was specifically designed and used for fast glucose analysis in blood samples from diabetics. Since then, the biosensor field has experienced important growth. In this context, amperometric glucose biosensors offer great potential for their use in the food production and processing industry. 

Amperometric glucose biosensors are prepared by immobilizing glucose oxidase (GOx) molecules onto an electrochemical interface. The enzyme catalyzes the conversion of glucose to gluconic acid and hydrogen peroxide. Glucose is quantified by the electrochemical measurement of hydrogen peroxide. One of the most important factors for the proper functioning of the biosensor is the correct selection of the electrochemical interface where the enzyme is immobilized. Recent researchers focused on the use of electrical conductors or semiconductor nanomaterials as biosensor interfaces [13,14]. The high surface area of nanomaterials allows immobilization of a large number of enzyme molecules, significantly increasing the sensitivity of the sensor [15]. Among all nanomaterials, titanium dioxide has been one of the most interesting materials in recent investigations [16,17] due to the facility to control the morphology of highly ordered titanium dioxide nanotube arrays (TiO_2_NTAs). Moreover, biocompatibility and ability to promote charge transfer processes make this material suitable as an electrochemical interface [18,19]. 

TiO_2_NTAs are formed by anodization from bare titanium [20,21,22]. In fluoride-containing electrolytes, the anodization of Ti is accompanied with the chemical dissolution of TiO_2_ due to the formation of TiF_6_^2−^ anions [23]. This formation process ends when TiO_2_ dissolution and formation rates reach the equilibrium. Due to the simplicity of the anodic formation as well as for all the TiO_2_NTAs properties, it has been extensively used as electrical interface for biosensor applications [14,17,18,24,25,26].

An important factor for the success of an enzymatic biosensor is the enzyme immobilization strategy. This process provides an intimate contact between the enzyme and the electrode surface. The objective is to maintain or improve the enzyme stability, properties, and the active structural conformation, as well as to allow the substrate to arrive at the active center of the enzyme [27]. Different physical and chemical immobilization techniques can be used to achieve this goal: surface adsorption, electrostatic interactions, covalent binding or polymer entrapment [28,29,30,31]. One of the simplest approaches is to entrap the enzyme within a gel layer, for example using Chitosan which is a biocompatible and biodegradable hydrogel [32,33,34] and contributes to stabilizing the enzyme molecules [31,35].

Several biosensor designs can be found in literature for glucose analysis in food stuff [36,37,38,39,40,41,42,43]. Usually, authors focus their efforts on new architectures to overcome matrix interferences or to modify the sensors’ linear range and sensitivity, as well as to keep the enzyme active as long as possible. However, there is a general lack of information in literature on other fundamental analytical parameters, like specificity, precision, accuracy, and robustness. These parameters must be deeply evaluated to prove both that a new measuring method or tool is capable of producing accurate results and to fulfill the quality assurance requirements of the food industry.

The goal of this work is to evaluate the analytical parameters of a biosensor to measure glucose in four different food products: soft drinks, soy sauces, dairy products and tomato sauces. The analyses have been performed using an amperometric glucose biosensor based on GOx immobilization with a polymeric hydrogel (Chitosan) onto highly ordered titanium dioxide nanotube arrays (TiO_2_NTAs). This sensor architecture was selected due to its simplicity and relative low cost.

## 2. Materials and Methods 

Titanium (99.7%, 5 mm diameter) was supplied by Alfa Aesar (Black Friel, MA, USA). Ethylene glycol (EG), ammonium fluoride and hydrogen peroxide were supplied by Panreac. Glucose, GOx (type: VII, *Aspergillus niger*, 100 units/mg) (ref. 101404648) and a low molecular weight Chitosan (ref. 1001654970) were supplied by Sigma Aldrich. The supporting electrolyte was 0.1 M pH 7.0 phosphate buffer solution (PBS).

The electrode morphology was characterized using a field emission scanning electron microscope (JEOL JSM-7001F, Tokyo, Japan). Linear sweep and amperometric measurements were performed in a standard three-electrode configuration (Ag/AgCl/3 M KCl was used as a reference electrode). All potentials mentioned in this work are referred to as the reference electrode. Experiments were performed using a potentiostat Autolab PGSTAT 302N and the working electrode was mounted in a rotating disc electrode system EG&G PARC model 616.

### 2.1. Synthesis of TiO_2_ Nanotubes Array

TiO_2_ nanotubes arrays were synthesized onto the Ti substrates by anodic oxidation. First of all, pure titanium disks were polished with SiC paper (2000 grit) and then were cleaned in ethanol prior to anodization. The cleaned Ti disks were anodized in a two-electrode electrochemical cell in ethylene glycol solution containing 0.3% NH_4_F and 2% H_2_O at 35 V for 17 h. This anodization time yields the maximum sensitivity of the sensor. Higher times result in the nanotube walls collapse. Then, the prepared electrode was sonicated during 30 s in water to remove surface debris. Finally, a thermal annealing was performed at 500 °C for 3 h in air to crystallize TiO_2_ nanotubes from amorphous to anatase phase [44].

### 2.2. Preparation of the Biosensor

**Figure 1:**
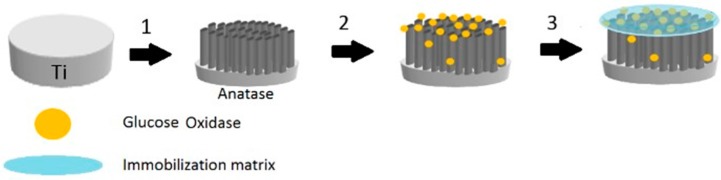
Scheme showing the construction of the glucose biosensor, step 1: growing and crystallization of titanium nanotubes; step 2: deposition of glucose oxidase molecules; and step 3: deposition of a polymeric cover (Chitosan) as immobilization matrix.

The immobilization of GOx on the modified TiO_2_ nanotubes array electrode (TiO_2_NTAs/Ti) was carried out by physical methods. An enzyme solution was prepared by dissolving 15 mg of GOx in 500 μL of 0.1 M PBS (pH 7.0). Then, the enzyme solution was immobilized onto the modified electrode using the hydrogel Chitosan (see Figure 1).

A 0.5% Chitosan solution was prepared by dissolving 53 mg Chitosan in 1% acetic acid. 20 μL of GOx solution and 20 μL of Chitosan solution were deposited on a TiO_2_NTAs/Ti electrode, then mixed and dried with an air stream. The obtained Chitosan–GOx/TiO_2_NTAs/Ti biosensor was washed with PBS to eliminate the enzyme that had not been immobilized. Finally, it was immersed in PBS solution for at least 30 min to rehydrate the enzyme molecules. When the electrodes were not in use, they were kept immersed in 0.1 M PBS (pH 7.0) at 4 °C.

### 2.3. Samples Preparation

Samples for the amperometric biosensor measurements did not need further preparations than dilution. For the soft drinks and the soy sauces, an adequate volume of the sample was directly added to the measuring chamber, containing 100 mL of 0.1 M PBS (pH 7.0). Dairy products and tomato sauces were first diluted into 50 mL of water. Then, an adequate volume of the diluted sample was directly added to the measuring chamber, containing 100 mL of 0.1 M PBS (pH 7.0). All samples were quantified by the standard additions method. 

### 2.4. HPLC and Amperometric Measurements

Glucose was determined by HPLC using an Agilent Technologies 1200 Series Chromatograph. Separation was done using a Kromasil^®^ 100 NH_2_ column with 5 μm of particle size, 250 mm longitude and 4 mm inner diameter. Elution was with 75% acetonitrile in ultrapure water. Eluted components were detected using a refraction index detector (Agilent G1362A) and quantified by direct interpolation in a calibration curve. 

Soft drink samples for the HPLC analysis were prepared by diluting 10 mL into 100 mL of ultrapure water and then were filtered through 0.45 μm nylon filters. Dairy products, soy and tomato sauces were prepared by dissolving an adequate amount of sample into 50 mL of Milli-Q water, followed by 10 min ultrasonication. The resulting mixture was centrifuged at 5000 rpm for 10 min. The supernatant solutions were filtered through 0.22 μm filters prior to injection.

Glucose was also quantified in the test sample using an amperometric/enzymatic titration method [45,46]. In this analytical method, GOx catalyzes the oxidation of glucose by dissolved oxygen to yield gluconic acid and hydrogen peroxide (Equation (1)). Then, hydrogen peroxide reacted with iodide to produce iodine in presence of molybdate as a catalyst (Equation (2)).

Glucose + O_2_ → H_2_O_2_ + Gluconic Acid (1)

H_2_O_2_ + 2I^−^ + 2 H^+^ → I_2_ + 2H_2_O (2)

The iodine formation is followed continuously by monitoring the current in a two-electrode system (Pt electrodes) by applying a constant potential of 100 mV. Under these conditions, the slope of the curve current vs. time is proportional to the glucose concentration. Then, a calibration curve can be constructed by plotting the slopes vs. the glucose concentration. This method has been thoroughly explained in the literature [45,46]. The glucose content in the test sample was determined by direct interpolation in the calibration curve.

## 3. Results and Discussion

### 3.1. Biosensor Construction and Determination of Its Optimal Working Conditions

**Figure 2:**
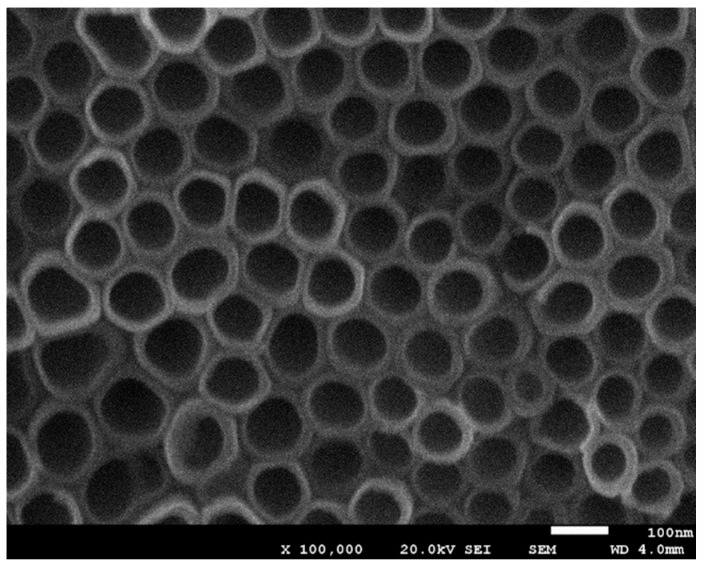
FE-SEM image of the formed TiO_2_ nanotube arrays.

The morphology of the titanium substrate after anodization was studied by FE-SEM analysis (JEOL JSM-7001F). Figure 2 shows the ordered tubular structures obtained after anodization.

FE-SEM image (Figure 2) revealed that highly ordered and vertically aligned TiO_2_ nanotube array was obtained during the anodization process. The formed nanotubes had an open-mouth structure on the top of the TiO_2_ layer, with estimated average dimensions of 80 nm inner diameter and 10 nm wall-thickness. The pore size and regular hollow structure stem from the anodic oxidation process [18]. 

The glucose biosensor was fabricated by immobilizing GOx using Chitosan onto TiO_2_NTAs/Ti. This sensor architecture was selected due to its simplicity and relative low cost, as well as for the high active area that allows immobilization of a high number of enzyme molecules. Moreover, Chitosan and anatase phase provide high biocompatibility to the system which can improve the long-term stability of the biosensor [18,19,31,32,33].

**Figure 3:**
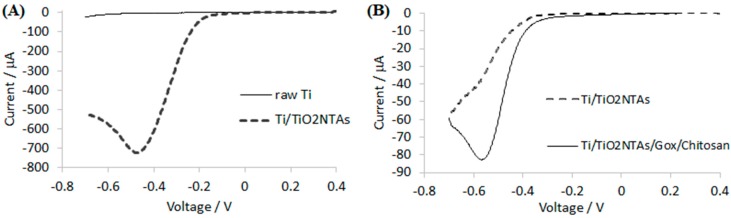
Linear sweep voltammograms of (**A**) Ti and Ti/TiO_2_NTAs sensors in PBS with 5 mM H_2_O_2_ and (**B**) Ti/TiO_2_NTAs sensor and Chitosan–GOx/TiO_2_NTAs/Ti biosensor in PBS with 5 mM glucose. Scan rate: 100 mV·s^−1^.

Glucose quantification was performed indirectly by reducing hydrogen peroxide (generated by the enzymatic reaction) to water at the electrochemical interface (TiO_2_NTAs/Ti). Two experiments were performed in order to evaluate H_2_O_2_ reduction over the TiO_2_NTAs/Ti electrochemical interface and glucose sensing with the Chitosan–GOx/TiO_2_NTAs/Ti biosensor. On one hand, TiO_2_NTAs/Ti response in PBS containing 5 mM of hydrogen peroxide was recorded and compared with bare titanium (see Figure 3A). On the other hand, Chitosan–GOx/TiO_2_NTAs/Ti biosensor response versus 5 mM of glucose in 0.1 M PBS was performed and compared with the TiO_2_NTAs/Ti (see Figure 3B).

As can be seen in Figure 3A, the reduction of H_2_O_2_ was registered in both cases at potentials lower than −0.2 V. Bare Ti shows a reduction peak at −0.47 V and −3.5 μA, while the Ti/TiO_2_NTAs shows a higher reduction peak at the same potential (−723.0 μA). The presence of the Ti/TiO_2_NTAs increases the substrate response by a factor of 200 approximately. The difference in peak current is due to two different factors: the increase of the active surface area of the electrode and the catalytic activity of TiO_2_ through H_2_O_2_ reduction [47].

In order to evaluate the working potential of the Chitosan–GOx/TiO_2_NTAs/Ti biosensor, linear sweep volammetries were performed in PBS containing 5 mM glucose (see Figure 3B). The Chitosan–GOx/TiO_2_NTAs/Ti biosensor was compared with Ti/TiO_2_NTAs. It can be seen in Figure 3B that the presence of the enzyme immobilized on the electrical interface showed glucose response at potentials more cathodic than −0.2 V. This electrochemical activity is related to the hydrogen peroxide reduction generated by the enzyme in the presence of glucose. The biosensor showed a cathodic peak current of −82.8 μA at −0.57 V. To select the working potential applied to the biosensor two considerations were required: the first was to assure H_2_O_2_ reduction to H_2_O at the electrochemical interface and the second was to prevent enzyme denaturalization. Taking into account these considerations, it was decided to set the working potential at −0.4 V vs. Ag/AgCl.

### 3.2. Analytical Parameters of Chitosan–GOx/TiO_2_NTAs/Ti Biosensor

The evaluation of the analytical quality parameters is essential to ensure that the methodology is accurate, specific, reproducible and robust over the specified range where an analyte will be determined [48]. Furthermore, the Food and Drug Administration (FDA) requires to determine these analytical parameters in order to guarantee the reliability of the analytical results [49].

First of all, the glucose content of a test sample (a commercial lemon soft drink) was determined using high-performance liquid chromatography (HPLC), amperometric/enzymatic titration [45,46] and Chitosan–GOx/TiO_2_NTAs/Ti biosensor. The goal of this initial step was to assure the biosensor measurement is in good agreement with other classical analytical techniques. Glucose was determined by HPLC by performing 2 replicate measurements. The obtained glucose concentration in the test sample was 0.14 M. Glucose was also quantified in the test sample using the amperometric/enzymatic titration method [45,46] and the obtained glucose concentration was 0.15 M. Finally, a Chitosan–GOx/TiO_2_NTAs/Ti biosensor was constructed to determine the glucose concentration of the test sample using the standard additions method; the current was continuously measured in 100 mL PBS at pH 7.0 to record the blank signal, then an adequate volume of the sample was added and finally two additions of 0.25 mM glucose standard solution were made. The obtained glucose concentration was 0.15 M.

To summarize, all glucose concentrations are shown in Table 1. In addition, the standard deviation (s) and the relative standard deviation (RSD%) of the measurements are included in the table.

As can be seen in Table 1, the glucose content using HPLC and the amperometric/enzymatic method were in good agreement with the result obtained using the biosensor. The glucose concentration determined by HPLC (0.14 M) showed a deviation of 6.4% with respect to the glucose content determined by the biosensor. Using the amperometric/enzymatic method this deviation was even smaller (1.6%). It can be seen that there is a good agreement between the determinations performed using the biosensor and the results obtained using HPLC and the amperometric/enzymatic method.

#### 3.2.1. Evaluation of the Linear Range, Limit of Detection (LOD) and Limit of Quantification (LOQ)

**Figure 4:**
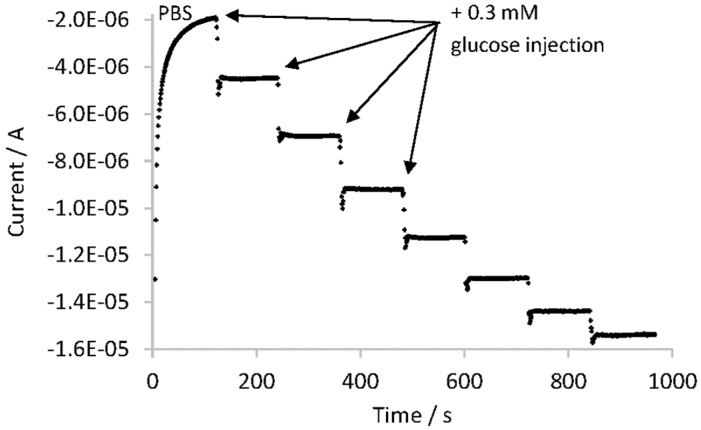
Current-time plot of Chitosan–GOx/TiO_2_NTAs/Ti biosensor with applied potential of −0.4 V when 0.3 mM glucose injections were done.

The amperometric response of Chitosan–GOx/TiO_2_NTAs/Ti biosensor to continuous injections of 0.3 mM glucose in 0.1 M PBS (pH 7.0) at −0.4 V vs. ref., was studied under a forced convection regime of the working RDE at 2000 rpm. Figure 4 shows the measured current over time after each glucose addition.

As can be seen in Figure 4, the registered current after each glucose addition (0.3 mM) increased approximately 2 μA. In order to determine the linear range of the Chitosan–GOx/TiO_2_NTAs/Ti biosensor, a calibration curve was built plotting the corrected current vs. the glucose concentration. The corrected current was calculated as the registered current after each glucose addition minus the blank signal (PBS). The obtained calibration curve had a slope equal to 5.46 μA·mM^−1^ and an intercept of 0.67 μA. A linear relationship of the corrected current to the glucose concentration was obtained between 0.3 mM and 1.5 mM glucose, with a correlation coefficient of 0.9902.

The slope of the calibration curve was associated to the biosensors sensitivity which is related to the limit of detection (LOD). The LOD was defined as the concentration that can be detected at 3 times the noise level and it was of 0.07 mM in glucose. This value is in good agreement with other LODs reported in literature [50,51] for similar glucose biosensors. In addition, the limit of quantification (LOQ) was also calculated, defined as the concentration that can be detected at 10 times the noise level and it was equal to 0.30 mM in glucose.

The linear range and sensitivity of the Chitosan–GOx/TiO_2_NTAs/Ti biosensor were compared with reported results of other glucose biosensors in literature (see Table 2). 

As can be seen in Table 2, the proposed biosensor achieved high sensitivity values (5.46 μA·mM^−1^), in comparison with other similar glucose amperometric biosensors reported in literature. This may result from the intrinsic structure of the Chitosan–GOx/TiO_2_NTAs/Ti electrode, which was able to retain a high number of enzyme molecules and conserve their active structure. On the one hand, TiO_2_ offers excellent biocompatibility and large active surface area to successfully immobilize a high number of GOx molecules. On the other hand, the Chitosan affinity to proteins prevents enzyme denaturalization, offering an enzyme friendly environment. Thus, high electrochemical activity was achieved due to the positive interaction of the electrochemical interface (TiO_2_NTAs) and the immobilization matrix hydrogel (Chitosan) with the enzyme.

When an analytical method is optimized, an increase in the sensitivity results in a shrinkage of the dynamic linear range [59]. In comparison with other reported biosensors in literature, the proposed biosensor has a smaller linear range (see Table 2), however it has a higher sensitivity (5.46 μA·mM^−1^). As a result, the linear range is smaller. It is worth mentioning that it is possible to control the sensor sensitivity on the suggested biosensor by decreasing the anodization time. It yields shorter nanotubes and as a result a smaller active area. Thus, in order to expand the linear range, the sensor sensitivity can be decreased by a diminution of anodization time. However, this factor was not considered in the present study.

#### 3.2.2. Evaluation of Precision: Repeatability and Reproducibility

In order to evaluate the repeatability of the method, five replicate measurements of the glucose content in the lemon soft drink sample were performed during the same laboratory session using the biosensor (see Table S1, Supplementary Materials). The average glucose concentration of the sample was 0.15 M, with a relative standard deviation (RSD%) equal to 0.8%. Taking into account that the RSD is lower than 1.9% (reference value suggested by the Association of Analytical Communities—AOAC [60]), it can be considered that measurements made with Chitosan–GOx/TiO_2_NTAs/Ti biosensor are repeatable.

Furthermore, sensor reproducibility was evaluated by measuring the glucose content of the test sample on five different days (see Table S2, Supplementary Materials). The average glucose content of test sample was 0.15 M with a 2.5% RSD. Therefore, this procedure also accomplishes the reproducibility requirements suggested by the Association of Analytical Communities—AOAC (<4%) [61].

#### 3.2.3. Evaluation of Accuracy

The accuracy of an analytical method is calculated as the percentage of recovery by the assay of the known added amount of analyte in the sample. It is necessary to perform at least nine determinations at three different analyte concentration levels [62]. The accuracy of the biosensor method was evaluated by adding 80%, 100% and 120% of the target concentration to a blank sample. For this purpose, a glucose free soft drink of the same type and brand of the analyzed test sample was used as a blank solution. Table 3 shows the recovery of the tested samples.

As it is seen in Table 3, the results were well agreed with the nominal glucose concentration values. The recoveries obtained of the prepared samples were comprised between 95% and 105% [63] (reference value suggested by the Association of Analytical Communities—AOAC).

Moreover, an additional set of recovery assays was performed: two known amounts of glucose were added to the sample to achieve a glucose concentration of a 120% and a 140% of the nominal concentration. Results of added samples are shown in Table 4.

Results in Table 4 show that the recovery rates were between 95% and 105% as in the previous situation. The satisfying results demonstrated that the Chitosan–GOx/TiO_2_NTAs/Ti biosensor was capable to determine glucose with an acceptable degree of accuracy.

#### 3.2.4. Evaluation of Biosensor Selectivity

**Figure 5:**
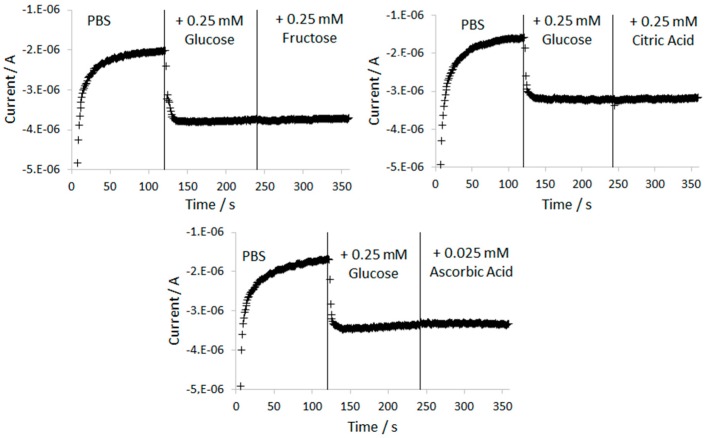
Amperometric responses of the Chitosan–GOx/TiO_2_NTAs/Ti biosensor measured in 0.1 M PBS pH 7.0 at potential of −0.4 V vs. Ag/AgCl with the addition of 0.25 mM glucose and 0.25 mM fructose (**A**); 0.25 mM citric acid (**B**) and 0.025 mM ascorbic acid (**C**) respectively.

In order to study the selectivity of the glucose biosensor, the effect of the common interfering substances was tested. Food samples, in particular fruit juices and soft drinks, contain different electrochemical active species that can modify the glucose signal. Some of the most common interferences are ascorbic acid, citric acid and other sugars such as fructose [36,64]. The effect of these three substances was studied in the selectivity test. The response of the Chitosan–GOx/TiO_2_NTAs/Ti sensor to sequential injections of 0.25 mM glucose and 0.25 mM fructose, 0.25 mM citric acid and 0.025 mM ascorbic acid in PBS solution was studied (see Figure 5). The ratio between the glucose and the interfering species concentration was previously selected according to its concentration in the test sample and the dilution factor in the quantification process.

As observed in Figure 5, when fructose, citric acid and ascorbic acid were added to the measuring solution, the current signals recorded by the biosensor were not affected. The biosensor exhibited strong response to 0.25 mM of glucose, approximately 1.6 μA, the studied interferences gave no significant amperometric responses under the working conditions.

Conventional amperometric glucose biosensors commonly work applying anodic potentials in order to oxidize H_2_O_2_ to oxygen. Then, the obtained anodic current will be proportional to the glucose concentration. Under these experimental conditions reductive species such as ascorbic acid, can be oxidized at the working electrode and thus the obtained current will be increased. Physical permselective barriers, such as Nafion, are commonly used in conventional glucose biosensors to block the access of this type of interfering species at the electrode surface [65]. This type of polymers may cause a loss of enzyme due to the instability of the enzyme in contact with the Nafion membrane [66]. As a result, the sensitivity of the analytical procedure will be decreased. However, in the Chitosan–GOx/TiO_2_NTAs/Ti biosensor the working principle is the H_2_O_2_ reduction to H_2_O using a cathodic potential. Under this condition, the change in the measured current is minimal due to the addition of ascorbic acid, as was previously demonstrated (see Figure 4), so it can be considered as a minor interference. In addition, it is worth mentioning that the biosensor constructed using a chitosan barrier offers an extra advantage which is the high sensitivity of the analytical procedure. This fact is explained due to the high biocompatibility and affinity of Chitosan to proteins, which favors the protection of the enzyme molecules [32].

#### 3.2.5. Evaluation of Robustness

Robustness is defined as the capacity of the analytical procedure to remain unaffected by small but deliberate deviations in the method parameters and provides an indication of its reliability during normal usage [49]. Robustness can also be described as the ability to reproduce the analytical method under different circumstances without the occurrence of unexpected differences in the obtained results. In this specific case, robustness refers to the entire process from the construction of the biosensor to the final glucose measurement.

In order to test the method robustness, five new biosensors were built; for each biosensor an anodization process was applied to obtain new and individual TiO_2_NTAs electrical interfaces, GOx and Chitosan solutions were freshly prepared for each individual biosensor and the glucose concentration in the sample was measured using each one of the five biosensors. Therefore, all the factors that could affect the fabrication of the biosensor were considered in the robustness test. These five new biosensors were used to quantify the glucose present in the sample over five different days (see Table S3, Supplementary Materials). The average glucose concentration obtained in the robustness test is 0.15 M with a RSD of 3.3%. This value indicates that the whole method, including the sensor construction and the measurement process is repeatable, reproducible, and robust.

#### 3.2.6. Evaluation of Storage Stability

Another important requirement of a biosensor is the long-term stability. Amperometric biosensors have commonly two disadvantages which affect their life time; enzyme leakage and enzyme denaturalization [67]. Therefore, the biosensor stability was evaluated by comparing the slopes of the calibration curves (related to the sensitivity) measured on different days. Table 5 shows the change in the signal of the Chitosan–GOx/TiO_2_NTAs/Ti biosensor during the stability test.

It can be seen in Table 5, Chitosan–GOx/TiO_2_NTAs/Ti biosensor retains an 85% of its initial sensitivity after 30 days from the first day measurement. This fact can be attributed to the excellent retention ability and good biocompatibility of the used immobilization matrix (Chitosan hydrogel). Chitosan forms a gel permeable to water with a high mechanical strength which, in combination with high affinity to proteins, favors the protection of the enzyme molecules [32]. Moreover, the obtained stability was similar to those of other glucose biosensors reported in literature which used Chitosan; after a month these biosensors retained between the 80% and the 90% of their initial response [33,34,68].

AOAC prescribes the minimum analytical performance requirements of analytical methods for the quality control of food products. The analytical criteria established by this organization are highly demanding. In this context, classical analytical techniques, such as HPLC or spectrophotometry, are usually evaluated and used to guarantee the quality standards of foodstuff. However, these analytical tools are time consuming and expensive. For this reason, the Chitosan–GOx/TiO_2_NTAs/Ti biosensor was evaluated as an alternative to these classical analytical methods. In this work, it was demonstrated that all analytical parameters tested for this biosensor were in good agreement with the AOAC quality standards. Furthermore, the measurement method in the biosensor analysis is faster and simpler than the conventional methods for glucose analysis. Therefore, the studied sensor had a great potential for practical application and can be considered as a low-cost, simple and rapid alternative to the classical methods to quantify glucose in food samples for quality control.

#### 3.2.7. Measurement of Glucose in Food Samples

The described Chitosan–GOx/TiO_2_NTAs/Ti biosensor was used to analyze the glucose concentration in four different alimentary products: soft drinks, soy sauces, tomato sauces and dairy products. The criteria selection of each sample matrix is to overcome interferences or quantification problems in classical techniques, such as HPLC. Soft drinks usually contain a high concentration of ascorbic acid and/or citric acid. Both species are classical interferents in the use of enzymatic methods. Soy sauces and tomato sauces are complex matrices with many elements in their composition that can influence the quantification process. It is worth mentioning that soy sauces present a high protein content and tomato sauces a high lipid content. Finally, dairy products were selected because of their high fat content. In addition, the presentence of lactose and galactose represents a common interference in the glucose determination using HPLC. In this study, the analyzed dairy samples were lactose free products. Therefore, this selection of samples depicts a large number of unfavorable situations for the glucose quantification in foodstuff. All selected samples were also analyzed by HPLC and the values obtained were considered as reference values. The results obtained with both analytical techniques are shown in Table 6.

As can be seen in Table 6, the glucose concentration of 20 different food samples with complex matrices was determined with sufficient precision using the biosensor. These results are in good agreement with those obtained by the HPLC method independently of the sample matrix. In all cases, the deviation between both methods was smaller than 10%. Therefore, the biosensor was able to overcome all possible interferences in the selected samples. In addition, the obtained deviation values were smaller than most of the reported values in literature [36,41,69,70]. Usually, reported deviations are equal or higher than 10%. Considering this and the short analysis time (approximately eight min per sample), the potential of the proposed biosensor for practical applications was demonstrated.

## 4. Conclusions

The analytical parameters of the Chitosan–Ox/TiO_2_NTAs/Ti biosensor were evaluated using a commercial lemon soft drink as a test sample. This biosensor showed a linear range from 0.3 mM to 1.5 mM of glucose with a low limit of detection (0.07 mM), low limit of quantification (0.30 mM) and high sensitivity (5.46 μA·mM^−1^). The measured glucose concentration of the sample showed a good agreement with other analytical techniques: the deviation from HPLC was 6.4% and from an amperometric/enzymatic titration 1.6%. Measurements done with the studied biosensor showed high repeatability (RSD equal to 0.8%), high reproducibility (RSD equal to 2.5%), high robustness and small analysis time (less than 10 min). In addition, the biosensor had good selectivity towards common interfering species in food matrices, such us ascorbic acid, citric acid and fructose. Finally, the storage stability was further examined and after 30 days, the GOx–Chitosan/TiO_2_NTAs biosensor retained 85% of its initial current response. 

The biosensor was used to determine the glucose concentration in four different types of alimentary samples (soft drinks, soy sauces, dairy products and tomato sauces). In all the cases, the glucose concentration was determined with sufficient accuracy (deviation less than 10%) regardless of the matrix composition. Therefore, it can be concluded that the electrochemical biosensor (Chitosan–GOx/TiO_2_NTAs/Ti) represents as a low cost, simple and rapid alternative to classical methods for glucose quantification in foodstuff.

## Figures and Tables

**Table 1:** Determination of glucose in a lemon soft drink using different analytical techniques.

Method	[Glucose]/M	s/M	RSD/%
HPLC	0.14	1.23 × 10^−3^	0.9
Amperometric	0.15	3.15 × 10^−3^	2.1
Proposed biosensor	0.15	2.02 × 10^−3^	1.3

**Table 2:** Some examples of glucose oxidase biosensors reported in the literature. The biosensor final application, sensitivity and linear range values are shown.

Schematic Biosensor Assembly	Sample Type	Linear Range/mM	Sensitivity/μA·mM^−1^	Reference
GCE/PB/PDA–GOx	Physiological samples	0.2–3.4	1.59	[52]
Gr/PANI/AuNPs/GOx	Whole blood	0.2–11.2	4.58	[53]
SPCE/PPy/CNC/GOx	-	1.0–20	0.73	[54]
GOx/PMPD–PB/GC	Serum samples	0.025–0.65	2.54	[55]
Cellulose/GOx/PB–SPCE	Beverages	0.25–2.00	2.14	[56]
GOx/AuPd/PI/RGO	Human serum	0.024–4.6	2.82	[57]
GA–GOx/Pt	Bioprocesses monitoring	0.01–20	1.47	[58]
Chitosan–GOx/TiO_2_NTAs/Ti	Food samples	0.3–1.5	5.46	Present work

AuNPs, gold nanoparticles; CNC, cellulose nanocrystal; GA, glutaraldehyde; GCE, glassy carbon electrode; Gr, graphene; PANI, polyaniline; PB, Prussian Blue; PDA, polymerized dopamine; PMPD, poly(m-phenylenediamine); PPy, polypyrrole; SPCE, screen printed carbon electrode.

**Table 3:** Recovery of glucose in prepared lemon soft drink samples.

Level	Nominal [Glucose]/M	Found [Glucose] ± s/M	Recovery/%
80%	0.120	0.122 ± 0.0015	102
100%	0.150	0.154 ± 0.0020	103
120%	0.180	0.187 ± 0.0027	104

**Table 4:** Recovery of glucose in added lemon soft drink samples.

Level	Added [Glucose]/M	Nominal [Glucose]/M	Found [Glucose] ± s/M	Recovery/%
120%	0.030	0.186	0.191 ± 0.0025	103
140%	0.060	0.216	0.227 ± 0.0018	105

**Table 5:** Study of variability between amperometric measurements done with Chitosan–GOx/TiO_2_NTAs/Ti biosensor on different days from their assembly.

Time/Days	Slope/μA·mM^−1^	R^2^	% Signal Decrease
0	5.46	0.9902	-
10	5.28	0.9902	3
20	4.69	0.9904	14
30	4.56	0.9903	15

**Table 6:** Determination of glucose in real food samples using the Chitosa—GOx/TiO_2_NTAs/Ti biosensor and a reference HPLC method. The deviation between both methods is also shown.

Type of Sample	Sample	[Glucose]_biosensor_ ± s/M	[Glucose]_HPLC/_M	Deviation/%
Soft drinks	D1 Orange	0.241 ± 0.008	0.251 ± 0.001	−4.1
	D2 Lemon	0.151 ± 0.002	0.141 ± 0.001	6.3
	D3 Lemon	0.176 ± 0.006	0.189 ± 0.001	−6.7
	D4 Orange	0.215 ± 0.019	0.224 ± 0.001	−4.0
	D5 Lemon	0.212 ± 0.011	0.209 ± 0.001	1.4
	D6 Orange	0.221 ± 0.010	0.232 ± 0.001	−4.4
	D7 Cola	0.156 ± 0.008	0.152 ± 0.001	2.3
	D8 Cola	0.177 ± 0.011	0.168 ± 0.007	5.2
Soy sauces	S1	0.520 ± 0.025	0.537 ± 0.026	−3.1
	S2	0.089 ± 0.005	0.096 ± 0.001	−7.3
	S3	0.250 ± 0.004	0.253 ± 0.009	−1.5
	S4	0.745 ± 0.003	0.775 ± 0.001	−3.8
	S5	0.093 ± 0.003	0.090 ± 0.001	2.7
Dairy products	L1 Milk	0.125 ± 0.005	0.134 ± 0.001	−7.1
	L2 Milkshake	0.170 ± 0.006	0.160 ± 0.001	6.1
	L3 Yoghurt	0.142 ± 0.006	0.138 ± 0.001	2.7
	L4 Yoghurt	0.125 ± 0.004	0.119 ± 0.001	5.4
Tomato sauces	T1 Fried Tomato	0.120 ± 0.001	0.111 ± 0.003	7.6
	T2 Fried Tomato	0.092 ± 0.003	0.088 ± 0.001	4.9
	T3 Ketchup	0.533 ± 0.004	0.515 ± 0.007	3.3

## Supplementary Materials

The following are available online at http://www.mdpi.com/1424-8220/17/11/2620/s1, Table S1: Study of the system repeatability. Measurements done during the same laboratory session; Table S2: Study of the system reproducibility. Measurements done during five days over five lemon samples; Table S3: Study of the method robustness. The measurements were done with five different Chitosan–GOx/TiO_2_NAs/Ti biosensors.
