# Supplementary material for: Analytical Parameters of an Amperometric Glucose Biosensor for Fast Analysis in Food Samples
Source: Sensors (Basel). 2017 Nov 14;17(11):2620. doi: 10.3390/s17112620 (PMC5713114; doi:10.3390/s17112620)
Supplement: Supplementary file 1 [file sensors-17-02620-s001.pdf]

Supplementary Materials

Table S1. Study of the system repeatability. Measurements done during the same laboratory session.

Replicate	[Glucose] /M	[Glucose] average / M	s / M	RSD / %
1	0.15			
2	0.15			
3	0.16	0.15	$1.19 x 10^{-3}$	0.8
4	0.15			
5	0.15			

Table S2. Study of the system reproducibility. Measurements done during five days over five lemon samples.

Sample	[Glucose] / M	[Glucose] average / M	s / M	RSD / %
1	0.15			
2	0.15			
3	0.14	0.15	$3.75 x 10^{-2}$	2.5
4	0.15			
5	0.15			

Table S3. Study of the method robustness. The measurements were done with five different Chitosan-GOx/TiO_2NAs/Ti biosensors.

Biosensor	[Glucose] / M	[Glucose] average / M	s / M	RSD / %
1	0.14			
2	0.15			
3	0.15	0.15	$4.82 x 10^{-3}$	3.3
4	0.14			
5	0.15			
